# Validity and Reliability of the Amharic Version of EORTC QLQ‐OG25 Among Esophagogastric Cancer Patients in Ethiopia

**DOI:** 10.1002/cnr2.70035

**Published:** 2024-10-18

**Authors:** Lidya Genene Abebe, Abigiya Wondimagegnehu, Eva Johanna Kantelhardt, Adamu Addissie

**Affiliations:** ^1^ Department of Preventive Medicine School of Public Health, College of Health Sciences, Addis Ababa University Addis Ababa Ethiopia; ^2^ Institute of Medical Epidemiology, Biometrics and Informatics Martin‐Luther‐University Halle‐Wittenberg Germany; ^3^ Department of Gynaecology Martin‐Luther‐University Halle‐Wittenberg Germany

**Keywords:** EORTC QLQ‐C30, EORTC QLQ‐OG25, esophagus and gastric cancer

## Abstract

**Background:**

Cancers of the stomach and esophagus are the fourth and sixth most common causes of cancer‐related deaths worldwide, respectively. Although various tools have been developed to assess the quality of life of patients with esophagogastric cancer, EORTC QLQ‐C30 and EORTC QLQ‐OG25 are the most used all over the world. However, they have not been validated in an Ethiopian context. Therefore, this study aimed to evaluate the psychometric properties of EORTC QLQ‐OG25 among Ethiopian patients with esophageal and gastric cancer.

**Methods:**

EORTC QLQ‐OG25 is a 25‐item tool with 10 single items and six symptom scales: Eating restrictions, reflux, dysphagia, odynophagia, discomfort and pain, and anxiety. The tool was translated into Amharic according to the EORTC forward‐backward translation protocol. To check its validity and reliability, a cross‐sectional study among 158 patients was conducted from March to May 2020 at Tikur Anbessa Specialized Hospital, Addis Ababa, Ethiopia. The psychometric properties of EORTC QLQ‐C30 and EORTC QLQ‐OG25 were assessed using multitrait scale analysis, known group validity, convergent validity, and divergent validity. Internal consistency was examined with Cronbach's alpha.

**Result:**

Eighty‐three (52.5%) of the participants were men; the median age was 50 years (IQR = 18 years). The overall item correlation alpha values ranged between 0.39 and 0.7. All item correlations within their scales were greater than 0.4. The correlation coefficients between all items and their own domain were greater than for other domains. The esophagogastric and core questionnaire correlation ranged from −0.65 to 0.62. The tool showed a significant difference between patients with good physical function and those with impaired physical function.

**Conclusions:**

The findings suggest that the Amharic version of EORTC QLQ‐OG25 is a valid and reliable tool among patients from Ethiopia with esophagus and gastric cancer. Therefore, we recommend that researchers and clinicians use the core tool together with the specific tool.

## 1 Introduction

Gastric cancer is responsible for over 1 million new cases and 769 000 deaths in 2020. It ranks fifth in incidence and fourth in mortality worldwide. Esophagus cancer was also responsible for 604 000 new cases and 544 000 deaths in 2020 [1]. It ranks seventh in incidence and sixth in mortality. In Ethiopia, both gastric and esophagus cancer are among the 15 most common cases with 4106 new cases and 3785 deaths in 2020 [2].

Cancer and its treatment have psychological and emotional effects [3]. In addition, treatments for gastrointestinal cancer, as well as the disease itself, have been shown to have a negative impact on patients' quality of life [3, 4]. Because of an increase in incidence and improvements in its treatment [5, 6], many cancer people tend to live for a longer period of time. However, most of these patients experience several types of symptoms, either due to the side effects of the treatments or from the progression of the disease itself [7]. Ineffective management of these symptoms can compromise a person's ability to perform daily tasks and also affect survival [8, 9, 10, 11]. As a result, cancer treatments have begun to include quality of life as one endpoint in the continuum of cancer care [12, 13]. Consequently, the Ethiopian Federal Ministry of Health issued a directive on treating troubling symptoms in cancer patients [14]. However, only a few studies on the health‐related quality of life of patients with cancer have been conducted in Ethiopia [7, 15]. These observational studies revealed that the health‐related quality of life scores of cancer patients were worse than those of the general population.

It is important to have a sensitive and validated quality‐of‐life assessment tool. Therefore, the EORTC groups developed a cancer‐specific quality‐of‐life assessment tool for esophagogastric cancer [16]. It is highly recommended to perform a validation study whenever a tool is translated into another language [17]. The cultural differences between Ethiopia and areas where the tools were developed and validated make it difficult to directly adopt and use the tool without performing validation [16, 18]. To our knowledge, EORTC QLQ‐OG25 has not been validated for patients with esophagogastric cancer in Ethiopia. Therefore, the present study aimed to assess the validity and reliability of the tool among Ethiopian patients with esophagogastric cancer.

## 2 Materials and Methods

### 2.1 Study Design, Area, and Participants

A cross‐sectional study was conducted at Tikur Anbessa Specialized Hospital (TASH) from March to May 2020. TASH is the largest referral and teaching hospital in Ethiopia and serves as the country's main comprehensive cancer center including radiotherapy. This oncology center has two radiation therapy machines, 36 inpatient beds, and 12 outpatient chemotherapy beds. Oncology services are also provided by seven clinical oncologists and 25 oncology nurses.

Sample size calculation for scaling analysis is recommended to be greater than 100 or five subjects per variable. Thus, the sample size was calculated based on the recommendations for scaling analysis [19]. As Amharic is the official working language and spoken among the majority of Ethiopians, a total of 158 pathologically confirmed patients with esophagogastric cancer aged 18 and above who were able to speak Amharic were included in the study.

### 2.2 Instrument

EORTC QLQ‐OG25 is a 25‐item module. It was developed to increase the sensitivity and specificity of the EORTC QLQ‐C30 tool. This module has six symptom scales and 10 single‐item symptoms. These scales are eating restrictions, Reflux, Dysphagia, Odynophagia, Discomfort and pain, and anxiety [16]. In addition to this tool, EORTC QLQ‐C30 and an interviewer‐administered structured questionnaire were used to examine individuals' sociodemographic and clinical characteristics. Initially, the tool was translated into Amharic according to the EORTC forward‐backward translation protocol and piloted on 10 people to identify questions that were difficult, unclear, or distressing. None of the questions were found difficult, confusing, or burdensome, and participants included in the pilot test were excluded from the actual study.

After completion of the pilot test, the actual data collection took place. The data were collected by two nurses who work in the oncology center of TASH. The data collectors received a 3‐day data collection training session by the principal investigator, with a focus on the purpose of the study and the content of the questionnaire.

### 2.3 Data Analysis

To determine the proportion and frequency of sociodemographic characteristics and clinical data, descriptive statistics were calculated and are presented in tables. The validity and reliability of EORTC QLQ‐OG25 were assessed for internal consistency, convergent validity, divergent validity, and known group validity. The correlation of the specific tool with the original tool was checked to see if the tools were designed to measure different things. A Cronbach alpha score of 0.70 or greater was considered adequate [19]. Convergent and divergent validity was determined by employing multitrait scaling analysis. We employed multitrait scaling analysis as a comparison method. The previous study assessed convergent and divergent validity using multitrait scaling [16]. Convergent validity is supported if the item domain correlation is at least 0.40. Divergent validity is satisfied if the value of correlation coefficients between the item and its own domain is higher than other domains. Known group validity was checked using the Mann–Whitney test to see whether the tool was able to detect differences between groups. Patients were categorized as having better (≥ 46.7) or worse (< 46.7) physical function based on the median value of the physical scale of the core EORTC QLQ‐C30 [16]. Curative and better physical functions were hypothesized to score lower for the symptom items and scales. The correlations between scales and single items of EORTC QLQ‐OG25 and EORTC QLQ‐C30 were determined using Spearman's correlation coefficient. A correlation coefficient between 0.40 and 0.6 was considered moderate; one greater than 0.6 was considered as strong [16]. All scales and items were converted to a score from 0 to 100 [20] and SPSS version 21 was used for all statistical analysis.

## 3 Results

### 3.1 Sociodemographic Characteristics of Respondents

A total of 158 participants with a median age of 50 years (IQR = 18) were included in this study. Eighty‐three (52.5%) of the respondents were male and 93 (58.9%) of them were married; 57 (36.1%) of the participants had no formal education, while 34 (21.5%) of the respondents had attended college. One‐third (53; 33.5%) of the participants were housewives and more than half (54.4%) of them were from Addis Ababa (Table 1).

**TABLE 1:** Sociodemographic characteristics of respondents at Tikur Anbessa Specialized Hospital, Addis Ababa, 2020.

Variable	Category	Frequency (*n* = 158)	Percent (%)
Sex	Men	83	52.5
	Women	75	47.5
Educational status	No formal education	57	36.1
	Primary education	43	27.2
	Secondary education	24	15.2
	College and above	34	21.5
Occupation	Farmer	23	14.6
	Employed	39	24.7
	Housewife	53	33.5
	Retired	11	7.0
	Student	11	7.0
	Merchant	21	13.3
Region	Addis Ababa	86	54.4
	Oromia	38	24.1
	Amhara	18	11.4
	Others^a^	16	10.1
Marital status	Married	93	58.9
	Single	28	17.7
	Widowed	20	12.7
	Divorced	17	10.8
Age	20–29	2	1.3
	30–39	31	19.6
	40–49	40	25.3
	50–59	47	29.7
	≥ 60	38	24.1

^a^ Others = Tigray, Southern Nations, Nationalities and Peoples', and Afar.

### 3.2 Clinical Characteristics of Respondents

Of the total of 158 patients included in this study, 70.9% and 29.1% of them were diagnosed with esophageal and gastric cancer, respectively. Most (80.4%) of the patients were diagnosed at an advanced stage of the disease and, as a result, three‐fourths of the respondents (75.9%) took treatments with palliative intent. Regarding the type of treatment received, almost equal proportions of esophagogastric patients received either chemotherapy alone (24.7%) or chemotherapy with radiotherapy (25.3%) (Table 2).

**TABLE 2:** Clinical characteristics of respondents in Tikur Anbessa Specialized Hospital, Addis Ababa, 2020.

Variables	Category	Frequency	Percent (%)
Esophagogastric cancer patients (*n* = 158)			
Cancer site	Esophagus	112	70.90
	Stomach	46	29.10
Type of treatment	Chemotherapy	39	24.70
	Chemotherapy and surgery	36	22.80
	Surgery and radiotherapy	19	12.00
	Chemotherapy and radiotherapy	40	25.30
	Radiotherapy	14	8.90
	Chemotherapy, radiotherapy, and surgery	10	6.30
Treatment intent	Curative	38	24.10
	Palliative	120	75.90
Cancer stage	Stage I	12	7.6
	Stage II	19	12.0
	Stage III	10	6.3
	Stage IV	117	74.1

### 3.3 Reliability of EORTC QLQ‐OG25

Cronbach's α coefficient ranged from 0.39 to 0.71. Internal consistency reliability for reflux reached the 0.7 criterion and for dysphagia, eating, pain and discomfort, and anxiety, it was above 0.6. However, Cronbach's alpha coefficient for Odynophagia was low (0.39) (Table 3).

**TABLE 3:** Cronbach's alpha value for six multi‐item scales of EORTC QLQ‐OG25 tool in Tikur Anbessa Specialized Hospital, Addis Ababa, 2020.

Scale name	Number of items	Cronbach's alpha coefficient
Dysphagia	3	0.67
Eating	4	0.60
Reflux	2	0.70
Odynophagia	2	0.39
Pain and discomfort	2	0.67
Anxiety	2	0.65

### 3.4 Convergent and Divergent Validity of Multitrait Scale Analysis for EORTC QLQ‐OG25

As the data did not fulfill the normality assumption, Spearman's rho was used to determine the correlation between the item‐own scale correlation and item‐other scale correlation. A *p*‐value less than 0.05 was considered significant. The item‐own scale correlations were over 0.4 for all scales. All scales' item‐own scale correlations were higher than item‐other scale correlations. This suggests that the convergent and divergent validity was fulfilled for all scales (Table 4).

**TABLE 4:** Convergent and divergent validity for EORTC QLQ‐OG25 scales in Tikur Anbessa Specialized Hospital, Addis Ababa, 2020.

Scale	Item number	Item‐ own scale correlation	Item‐other scale correlation	*p*
Dysphagia	31, 32, 33	0.69–0.84	0.16–0.52	< 0.05
Eating	34, 35, 36, 37	0.64–0.69	0.19–0.53	< 0.05
Reflux	38, 39	0.87–0.88	0.18–0.41	< 0.05
Odynophagia	40, 41	0.77–0.80	0.16–0.51	< 0.05
Pain and discomfort	42, 43	0.85–0.88	0.16–0.39	< 0.05
Anxiety	44, 45	0.85–0.87	0.16–0.52	< 0.05

### 3.5 Correlation Between EORTC QLQ‐C30 and OG25

As can be seen in Table 5, almost all correlations between EORTC QLQ‐OG25 items and scales and EORTC QLQ‐C30 functional scales were negative. The highest degree of correlation was noted between trouble with coughing and emotional scales. In addition, physical, role, emotional, cognitive, and social functions had correlations above 0.4 with eating, reflux, anxiety, dry mouth, body image, choking when coughing, trouble with coughing and talking, and eating with other scales and items. Conversely, none of the EORTC QLQ‐OG25 items and scales had a moderate correlation with the global quality of life scale.

**TABLE 5:** Correlation between EORTC QLQ‐OG25 and functional subscales of EORTC QLQ‐C30 in Tikur Anbessa Hospital, Addis Ababa, 2020.

EORTC QLQ‐OG25 scales and items	PF	RF	EF	CF	SF	QOL
Dysphagia	−0.3**	−0.26**	−0.30**	−0.34**	−0.40**	−0.01
Eating	−0.57**	−0.49**	−0.52**	−0.40**	−0.25**	0.03
Reflux	−0.44**	−0.34**	−0.44**	−0.31**	−0.30**	−0.07
Odynophagia	−0.29**	−0.17*	−0.24**	−0.26**	−0.36**	0.01
Pain and discomfort	−0.27**	−0.15	−0.33**	−0.29**	−0.22**	−0.03
Anxiety	−0.59**	−0.25**	−0.38**	−0.20*	−0.17*	0.01
Eating with others	−0.35**	−0.47**	−0.45**	−0.33**	−0.24**	0.06
Dry mouth	−0.49**	−0.39**	−0.56**	−0.28**	−0.43**	0.00
Trouble with taste	−0.12	−0.01	−0.05	−0.29**	−0.22**	−0.02
Body image	−0.42**	−0.41**	−0.55**	−0.36**	−0.38**	−0.02
Trouble swallowing saliva	−0.31**	−0.22**	−0.19*	−0.27**	−0.40**	0.04
Choking when swallowing	−0.43**	−0.41**	−0.40**	−0.43**	−0.42**	0.02
Trouble with coughing	−0.48**	−0.41**	−0.65**	−0.41**	−0.52**	0.17*
Trouble talking	−0.54**	−0.62**	−0.53**	−0.31**	−0.21**	0.06
Weight loss	−0.26**	−0.20*	−0.39**	−0.52**	−0.39**	−0.08
Hair loss	−0.12	−0.19*	−0.02	−0.19*	0.06	0.00

Abbreviations: CF, cognitive function; EF, emotional function; PF, physical function; QOL, quality of life; RF, role function; SF, social function.

* Correlation is significant at the 0.05 level.

** Correlation is significant at the 0.01 level.

The highest degree of correlation between EORTC QLQ‐OG25 and C‐30 symptom scales was observed between Fatigue and Trouble with Talking items (*r* = 0.62). Correlations between Fatigue and EORTC QLQ‐OG25 ranged from −0.11 to 0.62. It had correlations above 0.4 with eating, reflux, and body image. Correlations between nausea and vomiting and EORTC QLQ‐OG25 ranged from 0.18 to 0.48. Likewise, the correlation between pain and EORTC QLQ‐OG25 ranged from −0.04 to 0.55. Similarly, dyspnea had a moderate correlation (*r* = 0.47) with the trouble with the coughing item of EORTC QLQ‐OG25. Correlations of insomnia with EORTC QLQ‐OG25 ranged from 0.04 to 0.48 (Table 6).

**TABLE 6:** Correlation between EORTC QLQ‐OG25 and symptom subscales of EORTC QLQ‐C30, Addis Ababa, Ethiopia, 2020.

EORTC QLQ‐OG25 scales and items	FAT	NVO	PAI	DYS	INS	APT	CON	DIA	FIP
Dysphagia	0.09	0.3**	0.29**	0.35**	0.16*	0.24**	−0.02	0.29**	0.36**
Eating	0.54**	0.35**	0.55**	0.40**	0.45**	0.49**	0.34**	0.27**	0.23**
Reflux	0.53**	0.48**	0.50**	0.22**	0.48**	0.46**	0.47**	0.37**	0.24**
Odynophagia	0.40**	0.40**	0.40**	0.34**	0.24**	0.35**	0.21**	0.19*	0.31**
Pain and discomfort	0.24**	0.13	0.20*	0.29**	0.04	0.12	0.16*	−0.10	0.12
Anxiety	0.38**	0.23**	0.35**	0.4**	0.36**	0.31**	0.26**	0.11	0.10
Eating with others	0.26**	0.31**	0.29**	0.22**	0.40**	0.35**	0.27**	0.27**	0.20*
Dry mouth	0.35**	0.24**	0.38**	0.4**	0.39**	0.29**	0.33**	0.18*	0.35**
Trouble with taste	−0.11	0.18*	−0.04	0.12	0.11	0.07	−0.19*	0.34**	0.14
Body image	0.41**	0.45**	0.45**	0.29**	0.48**	0.35**	0.37**	0.40**	0.37**
Trouble swallowing saliva	0.07	0.40**	0.26**	0.19*	0.28**	0.18*	−0.04	0.34**	0.28**
Choking when swallowing	0.35**	0.42**	0.36**	0.35**	0.29**	0.28**	0.13	0.24**	0.42**
Trouble with coughing	0.36**	0.34**	0.47**	0.47**	0.48**	0.33**	0.22**	0.18*	0.44**
Trouble talking	0.62**	0.30**	0.52**	0.26**	0.47**	0.33**	0.40**	0.27**	0.17*
Weight loss	0.24**	0.29**	0.36**	0.29**	0.32**	0.3**	0.21**	0.33**	0.33**
Hair loss	0.20*	0.18*	0.13	0.07	0.16	0.22**	0.18*	0.09	−0.05

Abbreviations: APL, Appetite loss; CON, Constipation; DIA, Diarrhea; DYS, Dyspnea; FAT, fatigue; FIP, financial problems; INS, Insomnia; NVO, Nausea/Vomiting; PAI, pain.

* Correlation is significant at the 0.05 level.

** Correlation is significant at the 0.01 level.

### 3.6 Clinical Validity of EORTC QLQ‐OG25 Tool

Clinical validity was checked by comparing the scores of multi‐item scales and single items of EORTC QLQ‐OG25 among two distinct groups. Patients receiving curative care had significantly lower scores for the items and scales than patients receiving palliative care. As for the physical function, except for pain, discomfort, and trouble with taste items, all other scales and items discriminated against patients who had a better physical function and worse physical function. Patients with better physical function had lower scores for the symptom items (Table 7).

**TABLE 7:** Known group comparisons: Scales and items in EORTC QLQ‐OG25 for clinically distinct groups at Tikur Anbessa Hospital, Addis Ababa, 2020.

EORTC QLQ‐OG25	Treatment intent			Physical function		
	Curative	Palliative	*p*	Lower	Better	*p*
	*n* = 38	*n* = 120		*n* = 81	*n* = 77	
Dysphagia	93.68	75.01	0.03	90.23	68.21	0.00
Eating	96.59	74.09	0.01	102.52	55.29	0.00
Reflux	91.72	75.63	0.06	93.17	65.12	0.00
Odynophagia	88.66	76.60	0.15	88.57	69.96	0.01
Pain and discomfort	82.80	78.45	0.60	84.57	74.16	0.14
Anxiety	96.54	74.10	0.01	101.09	56.79	0.00
Eating with others	87.13	77.08	0.16	91.48	66.90	0.00
Dry mouth	92.82	75.28	0.03	93.28	65.01	0.00
Trouble with taste	91.32	75.76	0.06	84.93	73.79	0.11
Body image	91.53	75.69	0.05	94.10	64.14	0.00
Trouble swallowing saliva	85.82	77.50	0.31	88.84	69.68	0.01
Choking when swallowing	93.95	74.93	0.02	90.83	67.58	0.00
Trouble with coughing	90.76	75.93	0.01	93.18	65.11	0.00
Trouble talking	88.07	76.79	0.17	99.41	58.56	0.00
Weight loss	85.43	77.62	0.33	88.17	70.38	0.01
Hair loss	29.83	35.82	0.28	38.36	29.31	0.05

## 4 Discussion

In this study, the psychometric properties of the EORTC QLQ‐OG25 tool were evaluated, specifically its reliability, convergent, divergent, and known group validity were checked. The scales in the tool had alpha values greater than 0.7 except for dysphagia, eating, odynophagia, pain and discomfort, and anxiety scales. The item‐specific scale correlations were greater than 0.4 and the item‐other scale correlations were below the item‐specific scale correlations. In addition, the tool distinguished patients with good physical function from those with impaired physical function and patients receiving curative treatment from those receiving palliative care.

In the EORTC QLQ‐OG25 tool, internal consistency scores ranged from 0.39 to 0.7. Except for the Reflux scale, the alpha values for all scales were below 0.7. This is lower compared to the results of the Polish (0.79–0.91), Hindi (0.49–0.85)Mexican (0.70–0.83), and original studies (0.67–0.87) [16, 18, 21, 22]. Although the alpha values for these studies were greater than 0.7, the original study had an alpha value of less than 0.7 for the Reflux scale and the Hindi study had an alpha value below 0.7 for Odynophagia. The lower variability in our data could contribute to this difference.

The correlation between item‐own scale and item‐other scale correlations for EORTC QLQ‐OG25 items supported convergent and divergent validity for all scales. This finding is consistent with those of previous studies [16, 18, 21].

The known group comparison of EORTC QLQ‐OG25 was also evaluated. The groups were divided based on treatment intent and physical function. Dysphagia, eating, anxiety, dry mouth, body image, choking when swallowing, and trouble with coughing items and scales differentiated the groups. In contrast to the previous study conducted in Mexico [21], these items and scales showed more severe symptoms in patients receiving curative treatment. Physical function also differentiated the groups for all scales and items except for the items Pain and Discomfort and Trouble with Taste items. This could be explained by the smaller sample size of gastric patients in the current study since these items are more common in gastric cancer patients [23]. The correlation between EORTC QLQ‐C30 and OG25 was above 0.4 for some items and scales and below 0.4 for others. This finding is consistent with a study conducted in Mexico [21] and suggests that the core questionnaire and the esophagogastric tool capture different dimensions of quality of life and one tool cannot replace the other.

While some discrepancies have been observed between our findings and previous studies, these differences could be attributed to variations in study participants, such as the stage of cancer (early, advanced) or the primary site of the tumor (esophageal, gastric). As noted in the references and the original study, most prior research has been conducted outside of Africa. Therefore, our findings demonstrate the validity of the tool in countries like Ethiopia.

### 4.1 Strengths and Limitations of the Study

During the translation of the tool, every step of the EORTC translation procedure was followed. To increase the representativeness of this study, patients were included regardless of their place of residence, treatment, and disease stage. However, most patients were in end‐stage cancer, so we cannot generalize the findings to early‐stage cancer patients. In addition, most of the patients included in the study were patients with esophageal cancer. Therefore, we could not verify whether the tool distinguishes patients with gastric cancer from those with esophageal cancer.

### 4.2 Conclusions and Recommendations

The Amharic version of EORTC QLQ‐OG25 was translated and can be used as a reliable and validated tool for patients with esophagus and gastric cancer. Thus, we recommend that researchers and clinicians use the core questionnaire along with the disease‐specific modules while assessing health‐related quality of life among patients with esophagogastric cancer.

## Author Contributions

Lidya Genene Abebe, Abigiya Wondimagegnehu, and Adamu Addissie conceived the concept and methodology of the study. Lidya Genene Abebe, Abigiya Wondimagegnehu, and Adamu Addissie contributed to data analysis and interpretation. Lidya Genene Abebe drafted the manuscript. Lidya Genene Abebe, Adamu Addissie, Abigiya Wondimagegnehu, and Eva Johanna Kantelhardt revised the manuscript for important intellectual content. All authors approved the final version of the manuscript.

## Ethics Statement

Ethical clearance was obtained from the Addis Ababa University (AAU) College of Health Science, School of Public Health Ethical Review Committee Ethical Clearance Committee. Permission to use the questionnaires was obtained from the EORTC Research Group using an online form at https://qol.eortc.org/form.

## Consent

Informed written consent was given by all participants. This study was conducted in accordance with the Declaration of Helsinki.

## Conflicts of Interest

The authors declare no conflicts of interest.

## Data Availability

The data are available upon reasonable request from the corresponding author.
